# Orthopaedic surgeons’ ability to detect pathologic hip fractures: review of 1484 fractures reported to the Norwegian Hip Fracture Register

**DOI:** 10.1186/s13018-023-04336-w

**Published:** 2023-11-04

**Authors:** Anders Sund, Eva Dybvik, Jan-Erik Gjertsen

**Affiliations:** 1 Department of Orthopaedic Surgery, Haukeland University Hospital, Bergen, Norway; 2 Norwegian Hip Fracture Register, Department of Orthopaedic Surgery, Haukeland University Hospital, Bergen, Norway; 3 Department of Clinical Medicine, University of Bergen, Bergen, Norway

**Keywords:** Hip fracture, Pathological fracture, Proximal femoral metastasis

## Abstract

**Background:**

The proximal femur is the most common location of metastases in the appendicular skeleton. Data on pathologic hip fractures, however, are sparse despite it is the most frequently operated pathologic fracture. The aim of this study was to investigate the ability of orthopaedic surgeons to identify pathologic hip fractures in an acute setting and secondly to validate the underlying cause of the pathologic fractures reported to Norwegian Hip Fracture Register (NHFR).

**Methods:**

In the NHFR dataset between 2005 and 2019, we identified 1484 fractures reported to be pathologic possibly secondary to a malignancy. These fractures were thoroughly validated by reviewing X-rays, the patient journal, the operation description for date, side, why there had been suspicion of pathologic fracture, and implant choice. Pathology reports were reviewed once a biopsy had been performed. Based on this validation, information in the NHFR was corrected, whenever necessary.

**Results:**

Of the 1484 fractures possible secondary to malignancy, 485 (32.7%) were not a pathologic fracture. When reviewing the 999 validated pathologic fractures, 15 patients had a pathologic fracture secondary to a benign lesion. The remaining 984 patients had a pathologic fracture secondary to malignancy. The underlying diagnosis reported was corrected in 442 of the 999 patients. The true rate of pathologic hip fractures secondary to malignancy in our material was 0.8%, and most patients had underlying prostate (30%), breast (20%), or lung (17%) cancer.

**Conclusion:**

Orthopaedic surgeons in Norway failed to report correct data on pathologic fractures and the corresponding cancer diagnosis in an acute setting in many patients. The corrected data on pathologic fractures in the NHFR from 2005 to 2019 can now be a valid resource for further studies on the subject.

## Background

Skeletal metastases are a major problem for cancer patients with disseminated disease leading to pain and immobility, and proper treatment is of great importance for improving quality of remaining life [1]. The proximal femur is the most common location of metastases in the appendicular skeleton [2]. The significant mechanical stress during weight load results in pain and a high risk of pathologic fractures in this location [3].

Extensive research on osteoporotic hip fractures has resulted in several evidence-based guidelines for improving treatment [4]. The data on pathologic hip fractures, however, are sparse despite it is the most frequently operated pathologic fracture [5]. The Norwegian Hip Fracture Register (NHFR) has collected data and evaluated treatment of all hip fractures in Norway since 2005 [6]. Pathologic hip fractures are also reported to the NHFR, but as up to today, these fractures have been excluded from all studies. Registration of pathologic fractures is problematic since orthopaedic surgeons may have problems deciding whether a fracture is pathologic or not in the acute setting at the time of surgery. The exact diagnose can sometimes not be made until results from intra-operative biopsies are available, often several weeks after surgery. Consequently, there has been some uncertainty as to whether the underlying diagnose of a pathologic fracture reported to the NHFR is correct. The aim of this study was, by use of the NHFR dataset, to investigate the ability of orthopaedic surgeons to identify pathologic hip fractures in an acute setting and secondly to validate the reported underlying cause of the pathologic fractures reported to the NHFR from 2005 to 2019.

## Methods

The NHFR has collected data on hip fracture patients operated at Norwegian hospitals since 2005 [6]. Immediately after each primary operation and reoperation for hip fracture, surgeons complete a one-page paper form that is sent to the register. This form includes detailed patient information such as the unique 11-digit Norwegian personal identification number, age, sex, cognitive impairment, comorbidities (according to the American Society of Anaesthesiologists [ASA] classification), time of fracture, time of the start of surgery, type of fracture, type of surgery, fixation or hemiarthroplasty, duration of surgery, surgical approach, and type of implant (identified by catalogue numbers).

As up to now, the surgeons have reported a hip fracture as pathologic or not directly after surgery in the operating theatre, with a possibility for a free text remark of what type of pathologic fracture, without any subsequent follow-up question to verify the diagnosis. Thus, it has not been possible to correct an incorrectly reported pathologic fracture after further investigations.

When defining data quality in medical registries, the three most cited quality attributes are completeness, coverage, and accuracy [7]. The NHFR has been found to have high registration completeness (91%) and 100% coverage compared to the Norwegian Patient Registry (national administrative database) [8]. The accuracy of data in the NHFR has not yet been investigated.

**Fig. 1:**
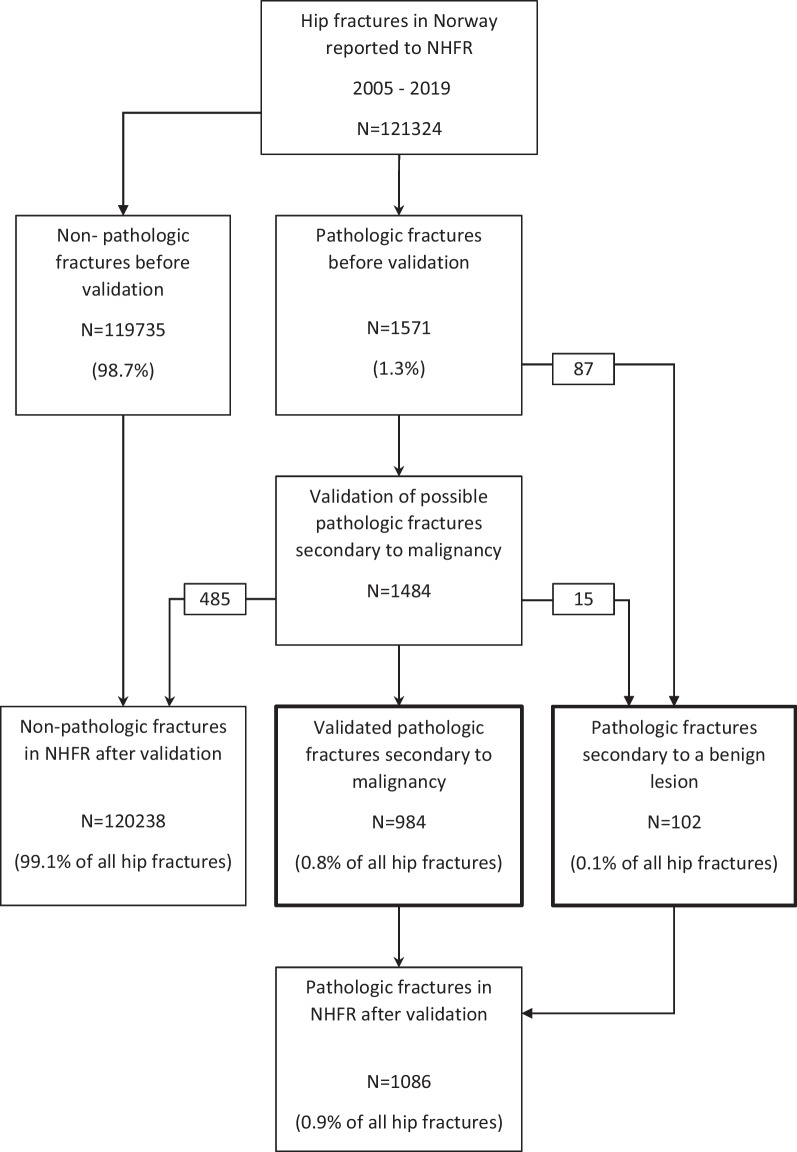
Flow sheet of study process

A retrospective study was conducted using prospectively collected data in the NHFR from 2005 to 2019. Of the 121,324 hip fractures in the NHFR, 1571 had been reported as pathologic fractures. These fractures were extracted for further analysis (Fig. 1). We divided the pathologic fractures into fractures reported to be secondary to a benign lesion, such as simple bone cyst, atypical femoral fracture, or giant cell tumour, and fractures reported to be secondary to malignancy, such as primary bone tumours or metastasis. Of the 1571 fractures, there were 87 fractures reported as secondary to a benign lesion (e.g. simple bone cyst, atypical femoral fracture, and giant cell tumour), the remaining 1484 fractures were possible fractures secondary to malignancy (primary bone tumour or bone metastasis). We wanted to investigate if these patients had a pathologic fracture due to malignancy and secondary to which type of lesion the fracture had occurred.

Each of the 1484 hip fractures was thoroughly validated by reviewing X-rays and the patient journal to access previous and actual cancer history, the operation description for date, side, why there had been suspicion of pathologic fracture, and implant choice. Pathology reports were reviewed once a biopsy had been performed. Earlier advanced imaging as CT scans, scintigraphy, and PET CTs was also reviewed to detect pathologic fractures not clearly visible on regular X-ray. Depending on the findings of the validation process, the fractures were divided into three groups: non-pathologic fracture (no signs of pathologic fracture); pathologic fracture secondary to a benign lesion (e.g. atypical femoral fracture or simple bone cyst); and pathologic fracture secondary to malignancy. The last group was defined by the presence of malignant cells on biopsy from fracture, lytic or/and sclerotic lesions on either X-ray, CT, or tumour on MRI in the presence of disseminated malignant disease. The systematic validation process was performed by a single orthopaedic surgeon specialized in orthopaedic oncology. Based on this validation, information in the NHFR was corrected, whenever necessary.

The project was approved by the Norwegian Regional Committee for Medical and Health Research Ethics (REK179521 southeast A) and the Data Protection Officer at each hospital. The NHFR is financed by the Western Norway Regional Health Authority. No competing interests were declared.

## Results

The results of the validation process are summarized in Table 1. Of the 1484 fractures possible secondary to malignancy, 485 (32.7%) were not a pathologic fracture. When reviewing the 999 validated pathologic fractures, 15 patients had a pathologic fracture secondary to a benign lesion. The remaining 984 patients had a pathologic fracture secondary to malignancy (Fig. 1). The diagnosis reported to the NHFR was corrected in 442 of the 999 patients.

**Table 1:** Summary of results of the validation process

Parameter	Number	Number corrected	Number correct
Pathologic fracture	1484	485 (not pathologic fracture)	999 (67.3%)
Diagnose	999	442 (corrected diagnose)	557 (55.8%)
Date primary surgery	999	3 (1 register form/2 punching data)	996 (99.7%)
Side	999	1 (1 register form)	998 (99.9%)
Implant type	999	0	999 (100%)
Date secondary surgery	999	0	999 (100%)

**Fig. 2:**
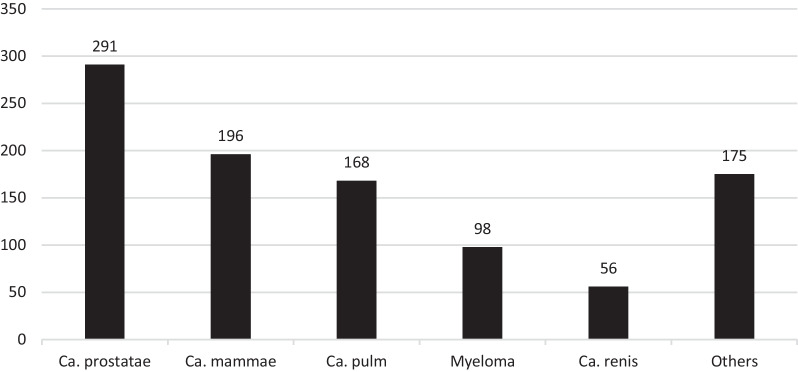
Distribution (numbers) of primary malignancies. Others include diagnoses < 5%

**Fig. 3:**
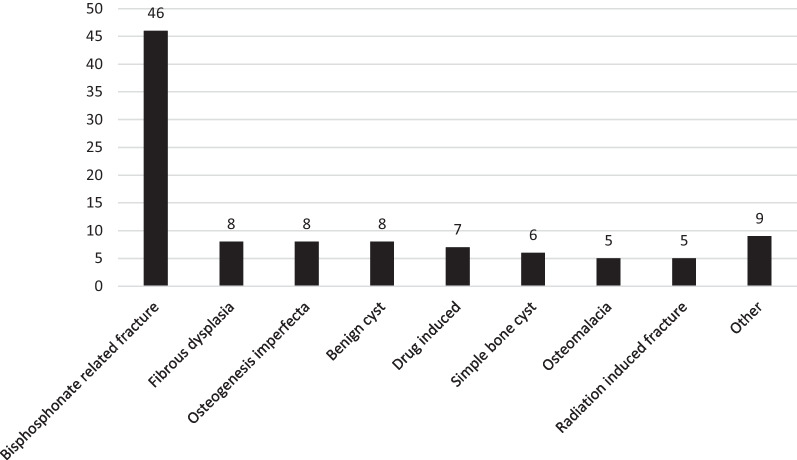
Distribution (numbers) of primary benign lesions. Other include diagnoses < 5%

In addition, the date of primary surgery was corrected in three cases. One was misreported on the form from the surgeon, and two were incorrectly transferred from the paper form to the NHFR database. In one form, the surgeon had reported a fracture on the wrong side. All reported data on implants and date of secondary surgery were correct (Table 1). The diagnoses of the 984 pathologic fractures secondary to a malignancy are listed in Table 2. Prostate cancer, breast cancer, and lung cancer were the dominating malignant diagnoses. These three diagnoses alone accounted for almost two-thirds of the malignant pathologic fractures (Fig. 2). Only one patient had a bone sarcoma. The pathologic fractures secondary to a malignant disease accounted for 0.8% of all fractures registered in the NHFR. Pathologic fractures secondary to a benign lesion accounted for 0.1% of all fractures registered in the NHFR and are listed in Table 3 and Fig. 3. Almost half of these fractures were atypical femoral fractures related to bisphosphonate treatment. Thus, atypical femoral fractures accounted for 0.04% of all fractures in the NHFR.

**Table 2:** Summary of malignant diagnosis related to pathologic fracture

Fracture secondary to malignant lesion	No. of 984	%
Ca. prostatae	291	29.6
Ca. mammae	196	19.9
Ca. pulm	168	17.1
Myeloma	98	10.0
Ca. renis	56	5.7
Ca. coli	26	2.6
Malignant melanoma	17	1.7
Ca. vesicae	16	1.6
Ca. pancreatic	12	1.2
Ca. origo incerta	11	1.1
B-cell lymphoma	10	1.0
Ca. oesophagi	10	1.0
Others *	73	7.4

*All malignant diagnoses below 1%

**Table 3:** Summary of benign diagnosis related to pathologic fracture

Fracture secondary to benign lesion	No. of 102	%
Bisphosphonate related	46	45.1
Fibrous dysplasia	8	7.8
Osteogenesis imperfecta	8	7.8
Benign cyst	8	7.8
Drug induced	7	6.7
Simple bone cyst	6	5.9
Osteomalacia	5	4.9
Radiation induced	5	4.9
Osteochondroma	4	3.9
Osteomyelitis	3	2.9
Fibromatosis	1	1.0
Stress fracture	1	1.0

## Discussion

This study showed that one out of three pathologic fractures in the Norwegian Hip Fracture Register had been incorrectly reported as pathologic. The underlying cancer diagnosis was incorrectly reported or missing in almost half of the cases.

After validation, 0.8% of fractures registered in the NHFR were pathologic fractures secondary to a malignant disease. Pathologic fractures secondary to a benign lesion were less common (0.1%). Prostate cancer (30%), breast cancer (20%), and lung cancer (17%) were the most common malignant diagnoses in our material. In a systematic literature review including 40 studies with a total of 3211 metastatic lesions in the complete femur, breast (35%), lung (15%), and prostate (10%) were the most common sites of primary tumour [9]. An observational study from the Swedish Fracture Register reported metastasis from prostate cancer most common (23%), followed by unknown (23%) and breast cancer (18%) in both operated and unoperated fractures of the lower extremity [10]. In an analysis of a large nationwide database from Japan, the most common primary sites of tumour were lung (19.2%), breast (16.6%), and prostate (10.3%) [11]. A large number of small patient series have shown a great variation in these numbers, but most of them report breast, prostate, and lung to be the most common primary tumours in patients with pathologic fractures [12–21]. In our material, in contrast with previously published studies, prostate cancer was the most common primary tumour. This difference may partly be a result of our complete population material, in contrast with other studies reporting on smaller selections of patients. In addition, differences in cancer incidences between different countries and parts of the world probably play an important role. The Norwegian cancer registry reports higher incidence for prostate cancer (178.6/100,000) than breast cancer (138.3/100,000) and lung cancer (119.5/100000), and higher mortality rate for prostate than breast cancer [22]. In contrast, the International Agency for Research on Cancer has reported a higher average rate for breast cancer (55.9/100,000) than for prostate cancer (37.5/100,000) and lung cancer (28.6/100,000) [23]. Rates from Japan show higher incidence of lung cancer and lower for prostate and breast cancer [24].

During validation of data in the NHFR, we observed a large number of patients who had been falsely reported to have a pathologic hip fracture. Other variables as date, side, and implant type had been reported and transferred from the form correctly.

As up to now, the surgeons have reported hip fractures to the NHFR by filling in a paper form, with no mandatory information on diagnosis or primary site of tumour. There has not been a uniform or clear recommended way of how to report pathologic fractures leading to some register data of low value. It is clearly difficult for the orthopaedic surgeon to decide if a fracture is for sure pathologic or not in the acute setting at time of surgery. The surgeon can suspect that a fracture may be pathologic secondary to a malignant disease based on examination of preoperative X-rays or CT scans or based on findings during surgery. In some cases, the patient has a known cancer diagnosis. The exact diagnose of disseminated disease can sometimes, however, not be made until results from intra-operative biopsies are available, often several weeks after surgery. In other cases, a pathologic fracture can be the first sign of a malignant disease. These patients have often been investigated by CT scan prior to surgery in order to identify the primary malignant diagnose, but the exact diagnoses have not always been identified at time of surgery. As a consequence, there is normally some uncertainty as to whether the underlying diagnose of a pathologic fracture is correct and also difficult to decide the correct cancer diagnosis to report when the operation form to the NHFR is filled in. The high number of incorrectly diagnoses found in this study is, therefore, not surprising but shows the importance of validating data before further studies on pathologic fractures are performed using data from the NHFR. On the other hand, there are an uncertain number of pathologic fractures registered as normal, which we have not been able to detect/validate due to the large number of patients (*n* = 119,753).

The incidence of pathologic fractures secondary to cancer is uncertain, and large validated studies are missing. A retrospective cohort study from the Registry for Geriatric Trauma of the German Trauma Society (Deutsche Gesellschaft für Unfallchirurgie (DGU)) (ATR-DGU) 2016–2020 showed that 211 of 29,541 patients (> 70 years old) suffered from pathologic hip fractures corresponding to a rate of 0.7%. The register does not contain information regarding diagnosis, and the number can be to low due to age > 70 [25]. A retrospective review of the American College of Surgeons—National Surgical Quality Improvement Program (ACS-NSQIP) database from 2011 to 2017 showed a total of 67,548 patients of which 378 (0.6%) patients had a pathologic fracture, but does not contain information regarding the histologic diagnosis [26]. The results from both the German and the US studies correspond well to the portion of pathologic fractures secondary to a malignant disease found in our study.

Prevention of insufficient data quality through clear definitions, standard guidelines for collection, and adequate training and motivation of personnel is of great importance [7]. Since 2021, the NHFR has started to implement electronically registration of hip fractures where the surgeon can tick off various benign and malign lesions. This will hopefully reduce the number of forms with missing information on any underlying malignant diagnosis. However, the uncertainty on the diagnose at time of surgery remains. In future, as a consequence of this study, the NHFR is planning for a follow-up form, which should be sent to the surgeon 4–6 weeks after surgery in cases where a pathologic fracture has been reported, to validate the diagnose after further investigation and pathology reports.

The strength of this register-based study is the large number of included patients. We included all pathologic hip fractures from all (43) hospitals operating these fractures in Norway. All diagnoses were thoroughly validated by one single orthopaedic surgeon with highly specialized competence in orthopaedic oncology. Both patient journals, pathology reports, advanced imaging as CT scans, scintigraphy, and PET CTs were reviewed making the diagnosis as accurate as possible.

A large weakness in our study is the possibility that pathologic fractures may have been wrongly reported as a non-pathologic fractures. These fractures could, unfortunately, not be detected and validated as it would have been far too time-consuming to validate all the 119,753 fractures reported as non-pathologic. Accordingly, some pathologic fractures are for sure missing, making the true incidence of pathologic fractures higher than 0.8%.

## Conclusion

Orthopaedic surgeons in Norway fail to report correct data on pathologic fractures (32.7% of cases) and the corresponding cancer diagnosis (44.2% of cases) in many patients. The true rate of pathologic hip fractures secondary to malignancy in our material was 0.8%, and most patients had underlying prostate (30%), breast (20%), or lung (17%) cancer. The incidence was comparable to other large series, but the rates of primary cancer differ a bit, probably as a result of the different rates of primary cancers in the area of studies. The data on pathologic fractures in the NHFR from 2005 to 2019 have now been validated and corrected and can be a valid resource for further studies.

## Data Availability

The dataset generated and analysed during the current study is not publicly available due to the regulations of the Norwegian Data Protection Authority and the Norwegian personal protection laws. Also, publication of data obtained from the patient records may compromise patients’ privacy/consent. The data obtained from the NHFR that supports the findings of this study are available from Anders Sund (corresponding author) upon reasonable request and with permission of NHFR.
